# TGFBI Promotes Tumor Growth and is Associated with Poor Prognosis in Oral Squamous Cell Carcinoma

**DOI:** 10.7150/jca.29958

**Published:** 2019-08-27

**Authors:** Bing-jie Wang, Kun-ping Chi, Ru-ling Shen, Sai-wei Zheng, Yang Guo, Jian-feng Li, Jian Fei, Yuan He

**Affiliations:** 1 Department of Oral Medicine, School & Hospital of Stomatology, Tongji University, Shanghai Engineering Research Center of Tooth Restoration and Regeneration, Shanghai, 200072, China.; 2 Department of Stomatology, Ningbo Yinzhou People's Hospital, Zhejiang 315040, China.; 3 Department of Pathology, First people's Hospital of Yunnan Province, Yunnan, 650032, China.; 4 Shanghai Laboratory Animal Research Center, Shanghai 201203, China.; 5 School of Life Science and Technology, Tongji University, Shanghai 200082, China.

**Keywords:** TGFBI, OSCC, CRISPR/Cas9, transcriptional sequencing, proliferation, migration

## Abstract

**Purpose**: In a previous study, we found that transforming growth factor beta-induced (TGFBI) is a hub gene strongly associated with oral squamous cell carcinoma (OSCC), using gene chip meta-analysis and PPI network analysis. Thus, the present study was established to explore the role of TGFBI in the pathogenesis of OSCC and to define the underlying mechanisms.

**Methods**: The correlations between TGFBI expression and the clinicopathological features and prognosis of OSCC were analyzed. Then, TGFBI-knockout HSC-3 cell lines were constructed using the CRISPR/Cas9 system. Cell proliferation, migration, and invasion in vitro were determined by cell counting, CCK-8, colony formation, and Transwell assays. Moreover, a xenograft animal study was implemented to determine the tumorigenicity and metastatic ability associated with TGFBI in vivo. The genes and pathways differentially expressed after TGFBI knockout were determined using transcriptional sequencing and bioinformatics.

**Results**: TGFBI expression was significantly higher in OSCC than in normal tissue. Its high expression was also correlated with high stage and was predictive of poor prognosis, as we expected. Knockout of TGFBI inhibited cell proliferation and clone formation, and enhanced cell migration and invasion in vitro. Besides, the xenograft animal study showed that TGFBI knockout suppressed tumor growth and metastasis in vivo. Furthermore, transcriptome sequencing revealed that genes associated with cell proliferation, metastasis, and inflammatory responses exhibited a change of expression upon TGFBI knockout. GO and KEGG analyses indicated that the function of TGFBI is related to responses to bacteria and inflammatory responses.

**Conclusions**: TGFBI overexpression can promote OSCC and is associated with poor prognosis in OSCC patients. TGFBI knockout can inhibit cell proliferation and metastasis in vivo. TGFBI may alter cell responses to bacteria, which causes an imbalance in the immune inflammatory response and promotes the development of OSCC.

## Introduction

Oral squamous cell carcinoma (OSCC) is one of the most common malignant tumors in clinical practice 1. Annually, over 300,000 new cases of OSCC are detected globally and over 140,000 patients die from this disease 2. The 5-year survival rate of OSCC is lower than 50%, mainly because of the limited range of active drugs and therapeutic targets; moreover, the incidence of this disease is still rising 3. Many factors have been revealed to be strongly related to the pathogenesis of tumors. In recent years, it has been accepted that inadequately resolved chronic inflammation may increase the risk of cancer 4. Infection is also considered a driver of inflammation-related tumors, and it has been reported that approximately 20% of cancers worldwide are associated with microbial infection 5. A recent study showed that neutrophils significantly infiltrated into the OSCC microenvironment and increased OSCC invasion through a tumor necrosis factor (TNFα)-dependent mechanism 6. However, at present, the relationship among the oral microbiome, inflammation, and the development of OSCC and any mechanisms underlying this remain unclear.

In our previous study, we observed that the transformed growth factor-beta-induced gene (TGFBI) functioned as a key hub gene in a protein-protein interaction (PPI) network, as revealed by a meta-analysis; we also showed that high expression of TGFBI may promote OSCC 7. TGFBI, also known as BGH3, encodes an extracellular matrix protein 8. As a connective protein, which can bind integrin to extracellular matrix proteins, TGFBI plays an important role in embryonic development, cell proliferation, adhesion, migration, differentiation, and inflammation 9-11.

Many researchers have studied the role of TGFBI in different kinds of tumor, with the findings suggesting that it has diverse functions. Several studies showed that TGFBI was abnormally overexpressed and could act as a tumor-promoting gene in gastric cancer, colon cancer, bladder cancer, and esophageal squamous cell carcinoma 12-15. However, in mesothelioma, breast cancer, and lung cancer, the expression of TGFBI was significantly decreased, so it might act as a tumor suppressor gene 16-18. Moreover, Ween et al. showed that TGFBI plays dual roles in ovarian cancer. They found that it had low expression in ovarian cancer tissue, but was highly expressed in peritoneal cells. Low expression of TGFBI in ovarian cancer cells promoted tumor growth, whereas its high expression in peritoneal cells facilitated the migration of cancer cells 19. However, there is still only limited information about the role of TGFBI in the tumorigenesis of OSCC. Thus, the present study was established to explore the role of TGFBI in the development of OSCC, as well as its underlying mechanisms.

## Materials and Methods

### Validation of TGFBI expression using OSCC-related datasets obtained from the GEO and TCGA microarray databases

We downloaded four OSCC-related datasets from the GEO database: GSE23558 (27 OSCC, 5 normal), GSE30784 (167 OSCC, 45 normal), GSE25099 (57 OSCC, 22 adjacent normal tissue), and GSE37991 (40 OSCC, 40 normal). We also downloaded the dataset of head and neck squamous cell carcinoma (HNSCC) from The Cancer Genome Atlas project (TCGA, https://tcga-data.nci.nih.gov/tcga/) to analyze the relationship between the expression of TGFBI and the survival rate.

### Clinical characteristics of patients and patient samples

Paraffin-embedded tissue blocks of patients diagnosed with OSCC at the First People's Hospital of Yunnan Province, Yunnan, China, from 2008 to 2017 were used in this study. The OSCC diagnosis was histopathologically confirmed by two pathologists. All patients were treated with surgery at the above-mentioned hospital. None of the patients underwent preoperative treatment such as chemotherapy or irradiation prior to tumor resection. The data collected from all subjects included the gender, age, and clinical features of the tumor (e.g., histopathological grade, tumor size, status of lymphatic metastasis). All patients provided written informed consent and the study protocol was approved by the ethics committee of the Faculty of Medicine for Human Studies, School of Medicine, Tongji University. Tumor stage was classified based on the TNM classification of the UICC (2010) 20 and the degree of differentiation was diagnosed according to the grade classification system of the World Health Organization 21.

### Immunohistochemical staining

Paraffin sections were deparaffinized and rehydrated. Incubation with anti-TGFBI or anti-Ki67 (Abcam, Cambridge, UK) was performed at 4°C for 20 h, and then the sections were washed with PBS. After incubation with secondary antibody, IHC was performed with the AEC Peroxidase (HRP) Substrate Kit (Vector Laboratories, California, USA), in accordance with the manufacturer's instructions. An immunoreactivity score (IRS) system was used as previously described 22 for semi-quantitative analysis of immunohistochemistry. Two experienced pathologists, who were blind to the clinicopathological data of the patients, were invited to analyze the biopsies and score the immunoreactivity. Each section was semi-quantitatively scored for the percentage of immunoreactivity as follows: 0 (0% immunoreactive cells), 1 (1%-10% immunoreactive cells), 2 (11%-50% immunoreactive cells), or 3 (>50% immunoreactive cells). We also semi-quantitatively scored the staining intensity of each section as follows: 0 (negative), 1 (weak, +), 2 (intermediate, ++), or 3 (strong, +++). The total immunoreactivity score was calculated as the sum of these two scores. Based on this score, all samples were classified as having low (≤3) or high expression (>3).

### Generation of TGFBI-knockout OSCC cell lines using CRISPR/Cas9

The human OSCC cell line HSC-3 was kindly provided by Professor Qianming Chen, School of Stomatology, Sichuan University, and fulfilled the criteria for identification as short tandem repeat (STR). The cells were cultivated with DMEM containing 10% fetal bovine serum, 100 mg/mL penicillin, and 100 mg/mL streptomycin (GIBCO, Grand Island, NY, USA), and incubated at 37 °C in a humidified 5% CO_2_ atmosphere. SgRNAs targeting TGFBI conserved exon 3 (the ENST00000442011.6 transcript) were designed in accordance with the instructions from Feng Zhang's Lab (at http://crispr.mit.edu/); the sequences are shown in Table 1. The sgRNAs were cloned into the PX330 plasmid. The puromycin resistance gene was cloned into the PGK plasmid for screening. Then, the recombinant plasmids were transfected into the HSC-3 cells by Lipofectamine 3000 (Invitrogen, Carlsbad, CA, USA). The cells were screened with puromycin (1 μg/ml) for 3 days and diluted to pick up individual cell-forming clones. Isolated single clones were analyzed using RT-qPCR and Western blotting.

### RNA isolation and reverse-transcription quantitative PCR

Total RNA was isolated using Trizol reagent (Tiangen, Beijing, China), in accordance with the manufacturer's instructions, and first-strand cDNAs were synthesized with a TransScript One-Step gDNA Removal and cDNA Synthesis SuperMix kit (TransGen, Beijing, China), in accordance with its instructions. Reverse-transcription quantitative PCR (RT-qPCR) was performed on qTOWER 2.2 (Analytik Jena, Jena, Germany) using a TransStart Top Green qPCR SuperMix Kit (TransGen, Beijing, China) to quantify the mRNA level of genes. The primer sequences are shown in Table 1. The relative mRNA levels were calculated using the 2^-ΔΔCT^ method with reference to β-actin as a housekeeping gene. All measurements were performed in triplicate.

### Western blot assay

Proteins were extracted from cells and tissues with a RIPA buffer containing protease inhibitor cocktail (Selleck Chemicals, Texas, USA). Equal quantities of protein (50 μg) from each sample were separated on a 10% SDS-PAGE gel, and then transferred to nitrocellulose membranes. The blot membranes were sealed in 5% nonfat milk for 1 h, and incubated with anti-TGFBI antibody (Proteintech Group, Illinois, USA) and anti-β-actin antibody (Abcam, Cambridge, UK) for 4 h at room temperature. The membranes were incubated with the corresponding fluorescence-conjugated secondary antibody for 1 h. The images of western blotting were viewed using an Odyssey two-color infrared laser imaging system (LI-COR Biosciences, Odyssey, USA).

### Cell proliferation assay

Cells were seeded in six-well plates (1×10^5^ cells per well) and counted using a Beckman Vi-Cell XR Cell Viability Analyzer (Beckman, Brea, California, USA) every 24 h over a 4-day period. For the CCK-8 assay, cells were plated in 96-well plates (1x10^4^ cells per well). The number of cells in each well was measured indirectly every 24 h over a 4-day period with the Cell Counting Kit-8 (CCK-8) (TransGen, Beijing, China), in accordance with the instructions. In brief, the medium was replaced with 100 µl of mixed medium, which was mixed with CCK-8 and DMEM medium at a ratio of 1:9. After 2 h of incubation, the optical density (OD) value at a wavelength of 450 nm was measured using a Synergy2 Multifunctional Microplate Reader (BioTek, Vermont, USA).

### Clone formation assay

Cells (1000 cells/well) were plated into a 10-cm dish and cultured for 20 days. Then, the cells were fixed with 4% paraformaldehyde (Sigma, Germany) for 10 min and stained with 0.1% crystal violet (Beyotime, Shanghai, China) for 20 min. The OD value at a wavelength of 450 nm was measured after decoloring with 33% acetic acid (Sinopharm, Shanghai, China).

### Migration assay and invasion assay

The cell migration and invasion assays were performed in 24-well plates using Transwell assays in a Transwell chamber (Millipore, Massachusetts, USA) without (migration assay) or with (invasion assay) Matrigel coating (BD Biosciences, New Jersey, USA). A total 5 x 10^5^ cells were placed in the upper chamber, while DMEM medium with 10% FBS was added to the lower chamber. After 24 h of incubation, the cells remaining on the upper membrane were carefully removed and the cells that had crossed the membrane were fixed with 4% paraformaldehyde and stained with 0.1% crystal violet. Then, 33% acetic acid was added for decolorizing and the OD value at a wavelength of 570 nm was measured.

### Tumor xenograft study

The study was approved by the institutional animal ethics committee at Tongji University, China. For the in vivo growth assay, HSC-3, HSC-3KO1, and HSC-3KO3 cells were injected subcutaneously into 8-week-old female BALB/C nude mice (1×10^7^ cells/mice in 200 μl of DMEM). After 7 days of tumor growth, the tumor size was measured every other day with digital calipers for a total of 4 weeks. The volume of the tumor was calculated using the following formula: x^2^y/2 (x<y), where y represents length and x represents width. Tumors were also weighed and fixed in formalin for immunohistochemistry after the mice had been sacrificed. For the in vivo metastasis assay, 8-week-old female BALB/C nude mice were injected with 1 × 10^6^ HSC-3 cells or HSC-3KO1 cells through the tail vein. Mice were sacrificed at day 60 after injection.

### RNA-seq and data analysis

Total RNA was isolated using Trizol. Samples were then submitted to Majorbio Biotechnology Co., Ltd., for RNA-seq analysis using the Illumina HiSeq platform. The sequence data were first mapped onto human reference genome sequences (hg19) with the TopHat (v v2.0.12) package. The Rsubread (v1.16.1) package was used to assign the mapped reads onto human refseq gene annotation (hg19) and reads per kilobase per million mapped reads (RPKM) was calculated, to quantify the gene expression level. The human reference sequence and the refseq gene annotation were downloaded from the UCSC website (http://genome.ucsc.edu). Differentially expressed genes (DEGs) were identified with the limma (v3.28.21) package with cut-offs of p < 0.05 and fold change >1.5. The heat map was made with cut-offs of p < 0.01 and fold change >3. GO biological process and KEGG pathway enrichment analyses for these DEGs were performed with the clusterProfiler (v3.0.5) package. The cluster analysis was performed with the pheatmap (v1.0.8) package.

### Statistical analysis

Data were analyzed using SPSS 20.0 software (SPSS Inc., Chicago, IL, USA). The *t*-test was used to analyze differences in gene expression. The chi-squared test was used to analyze the clinicopathological variables. One-way analysis of variance (ANOVA) was performed for the cell experiments. *P* < 0.05 was considered to represent statistical significance.

## Results

### High expression of TGFBI in OSCC predicts poor prognosis

**Fig 1:**
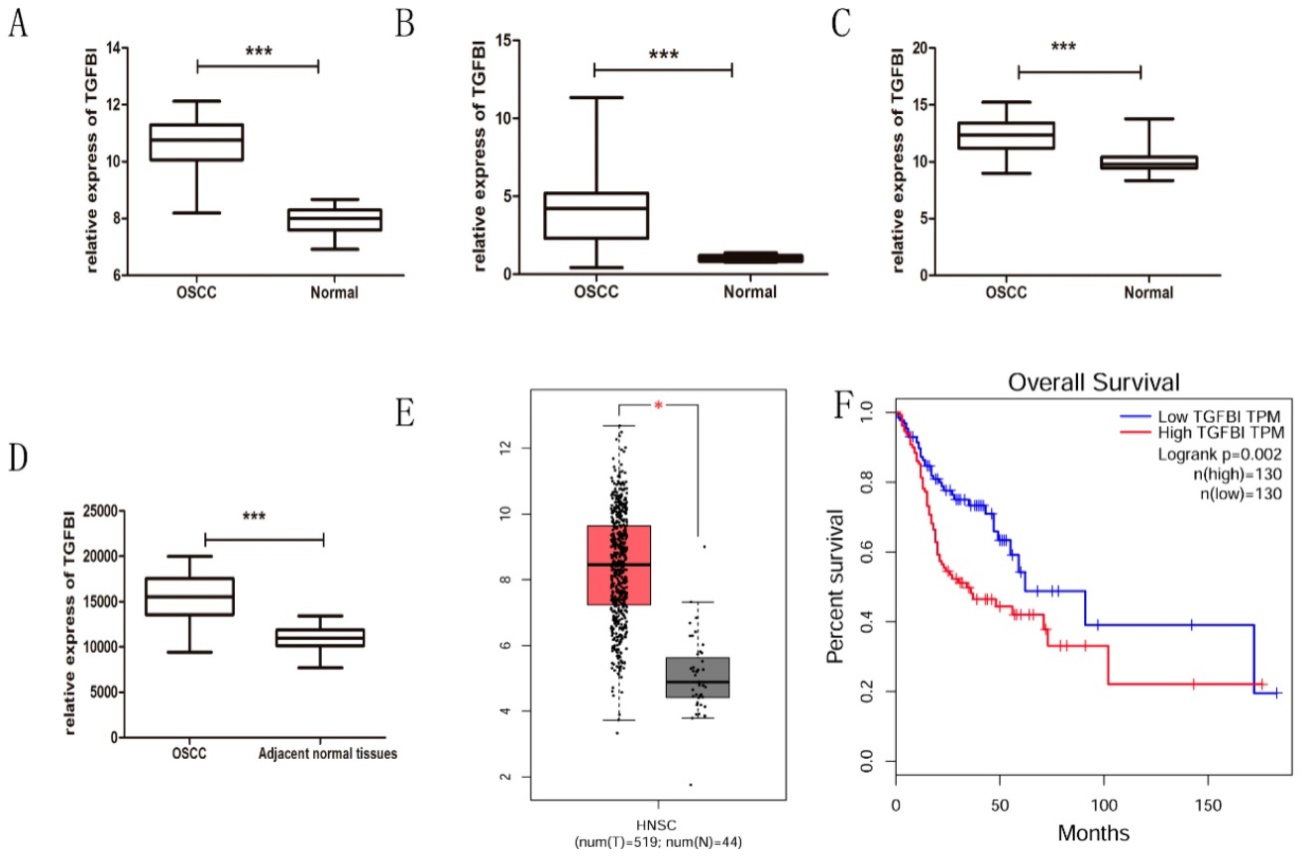
** TGFBI expression is significantly higher in OSCC tissue, as analyzed using GEO and TCGA databases.** The expression of TGFBI in OSCC was higher than in normal tissue, as analyzed using the GEO database (A) GSE23558, (B) GSE30784, (C) GSE25099, (D) GSE37991, and TCGA database (E: left: HNSCC, right: normal). The 5-year survival rate of HNSCC patients with high TGFBI expression was lower than that of patients with low expression, as revealed by Kaplan-Meier survival curve analysis (F).

After integrating the data from the GEO and TCGA databases, we confirmed by *t*-test that the expression of TGFBI in the OSSC tissue was significantly higher than that in the normal tissue (Fig. 1A-E), which was consistent with our previous study 7. Meanwhile, the Kaplan-Meier curves for HNSCC patients with a high or low TGFBI mRNA level were created according to the HNSCC dataset from the TCGA database (Fig. 1F). The survival rate of HNSCC patients with high TGFBI expression was lower than that of those with low TGFBI expression. Next, we analyzed the characteristics of the 52 patients with OSCC, the results of which are summarized in Table 2. High TGFBI expression in OSCC was significantly associated with the T classification (*P*=0.037) and a higher clinical stage (*P*=0.007). The above results suggested that the overexpression of TGFBI was associated with the development and poor prognosis of OSCC.

### TGFBI knockout inhibits proliferation of OSCC cells in vitro

**Fig 2:**
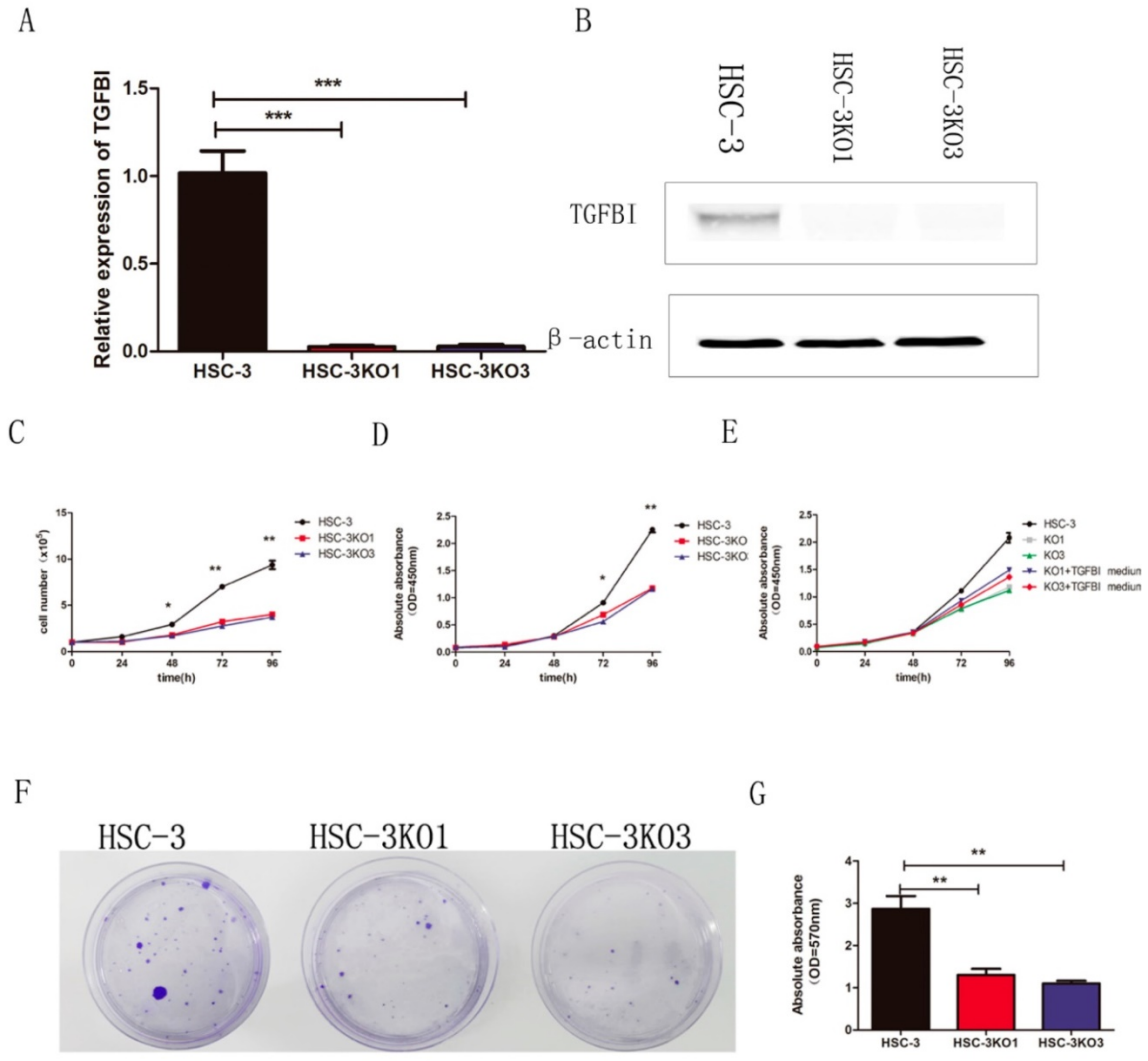
** TGFBI knockout inhibits proliferation of OSCC cells in vitro.** There was little TGFBI expression in HSC-3KO1 and HSC-3KO3, as revealed by RT-qPCR (A) and Western blotting (B). HSC-3KO1 and HSC-3KO3 cells grew significantly more slowly than HSC-3 cells, as revealed by cell count assay (C) and CCK-8 assay (D). The proliferation rate of HSC-3KO1 and HSC-3KO3 cells was increased after culture with the conditioned medium with TGFBI protein from HSC-3, as shown by CCK-8 assay (E). Colony formation ability was reduced in HSC-3KO1 and HSC-3KO3 cells compared with that of HSC-3, as shown by cell colony assay (F). Indirect quantification of cell colony assay results was performed by calculating the OD value at a wavelength of 570 nm after decoloring (G).

The sgRNA was cloned into a plasmid and transfected into the HSC-3 cells. We finally obtained two TGFBI-knockout cell clones and named them HSC-3KO1 and HSC-3KO3. RT-qPCR and western blotting showed that there was little TGFBI expression (Fig. 2A, B). Thus, we used these stable cell lines for further study.

In the cell proliferation assays in vitro (cell count and CCK-8 assays), both HSC-3KO1 and HSC-3KO3 cells grew significantly more slowly than HSC-3 cells (Fig. 2C, D). In addition, the proliferation rates of HSC-3KO1 and HSC-3KO3 cells were increased after culture with the conditioned medium from HSC-3 (Fig. 2E). Cell colony assays showed that the clone numbers and OD values of HSC-3KO1 and HSC-3KO3 cells were significantly decreased compared with those of HSC-3 (Fig. 2F, G). Taken together, these results suggest that TGFBI positively regulates OSCC cell growth and that TGFBI knockout inhibits the proliferation of HSC-3 cells.

### Inactivation of TGFBI promotes cell migration and invasion in vitro

**Fig 3:**
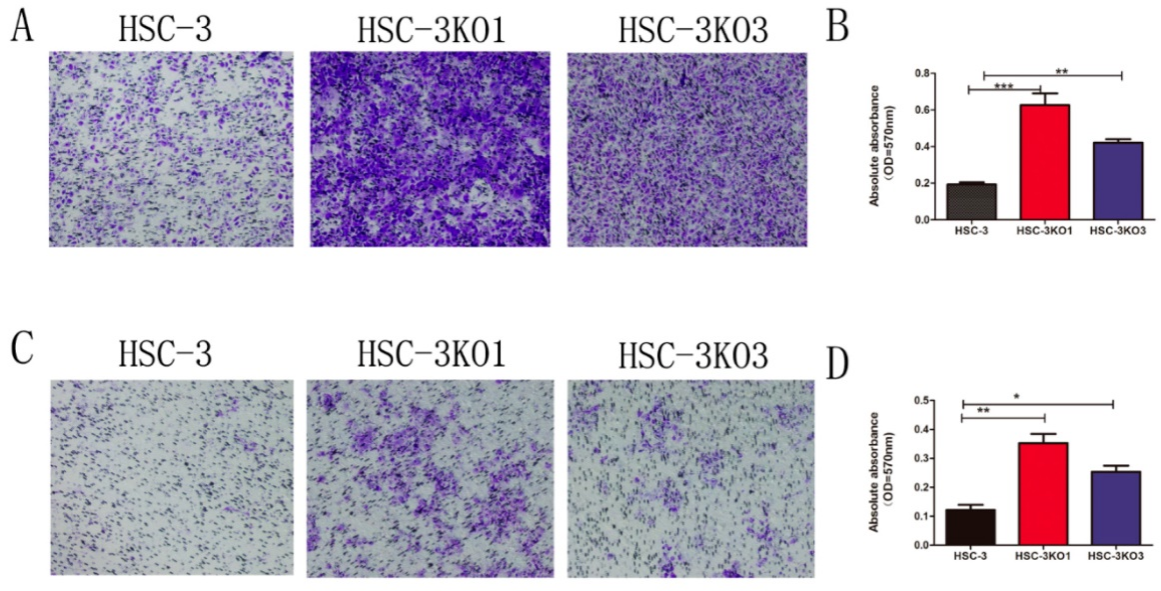
** Inactivation of TGFBI promotes cell migration and invasion in vitro.** The migration (A) and invasion (C) of HSC-3KO1 and HSC-3KO3 were clearly increased, as shown by Transwell assays without or with Matrigel coating. (B, D) were indirectly quantified results obtained by measuring the OD value at a wavelength of 570 nm after decoloring.

Compared with those of HSC-3 cells, the migration and invasion of HSC-3KO1 and HSC-3KO3 were significantly increased, as revealed by Transwell assays (Fig. 3A, C). After decolorizing, the OD values of HSC-3KO1 and HSC-3KO3 were also significantly higher (Fig. 3B, D). These results indicated that the inactivation of TGFBI could enhance cell migration and invasion in vitro.

### Knockout of TGFBI suppresses tumor growth in vivo

**Fig 4:**
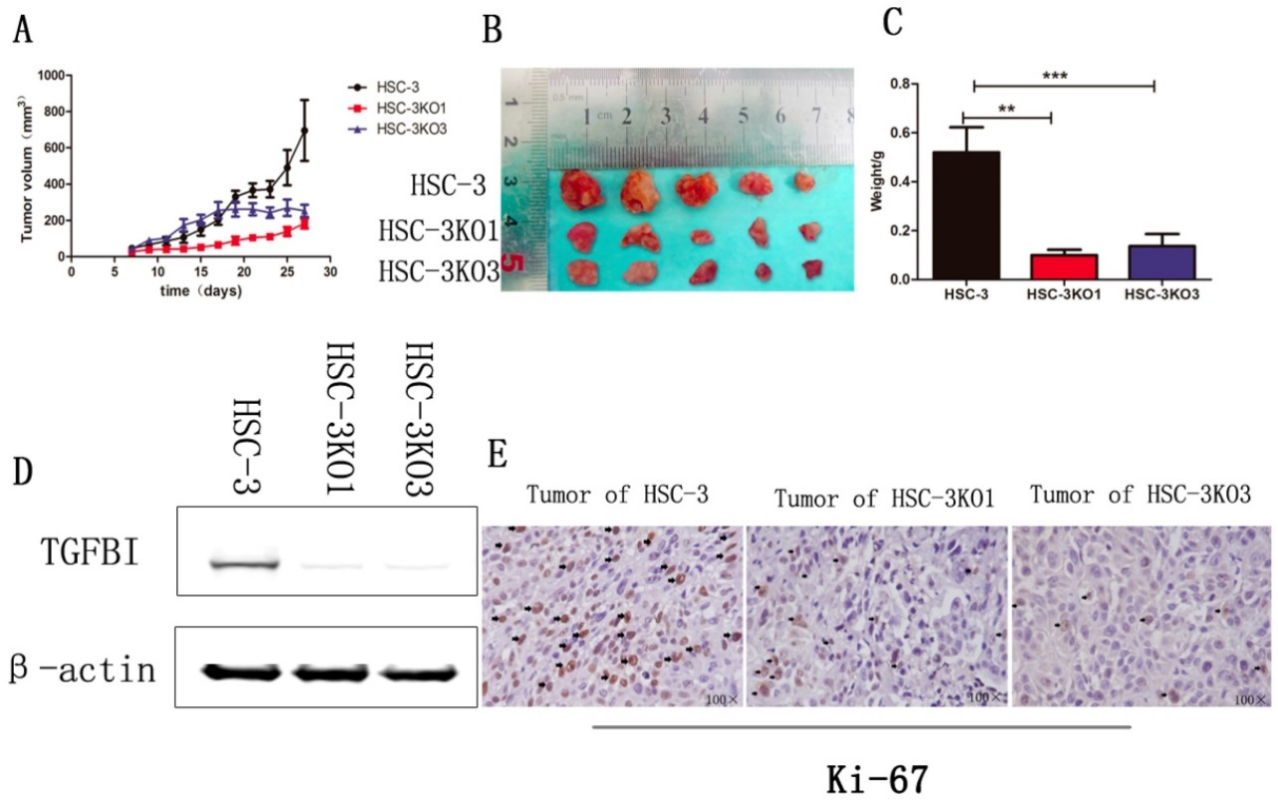
** Knockout of TGFBI suppresses tumor growth in vivo.** The HSC-3KO1 and HSC-3KO3 cell-generated tumors grew more slowly than those generated by HSC-3 cells, as shown by xenograft animal study (A). The volume (B) and weight (C) of xenograft tumors from HSC-KO1 and HSC-3KO3 were significantly reduced compared with those from HSC-3 cells after 4 weeks of tumor growth. The protein expression of TGFBI in tumors from HSC-3KO1 and HSC-3KO3 was significantly decreased, as shown by Western blot (D), and the expression of Ki-67 was decreased in HSC-3KO1 and HSC-3KO3 cell-generated tumors, as shown by immunohistochemical staining (E). Some of the Ki-67-positive cells are indicated by black arrows.

The effect of TGFBI on the growth of tumors in vivo was evaluated using a xenograft animal study. Cells were injected subcutaneously into BALB/C nude mice. The tumors generated from HSC-3KO1 and HSC-3KO3 cells grew slower than those generated from HSC-3 cells. The volume (Fig. 4A), size (Fig. 4B), and weight (Fig. 4C) of tumors generated from HSC-3KO1 and HSC-3KO3 cells were significantly reduced compared with those from HSC-3 cells after 4 weeks of tumor growth. The protein expression of TGFBI in tumors from HSC-3KO1 and HSC-3KO3 was also significantly decreased, as shown by western blotting (Fig. 4D), as was the expression of Ki-67, as revealed by immunohistochemical staining (Fig. 4E). The above results indicated that TGFBI knockout could suppress OSCC tumor growth in vivo.

### Knockout of TGFBI suppresses tumor metastasis in vivo

**Fig 5:**
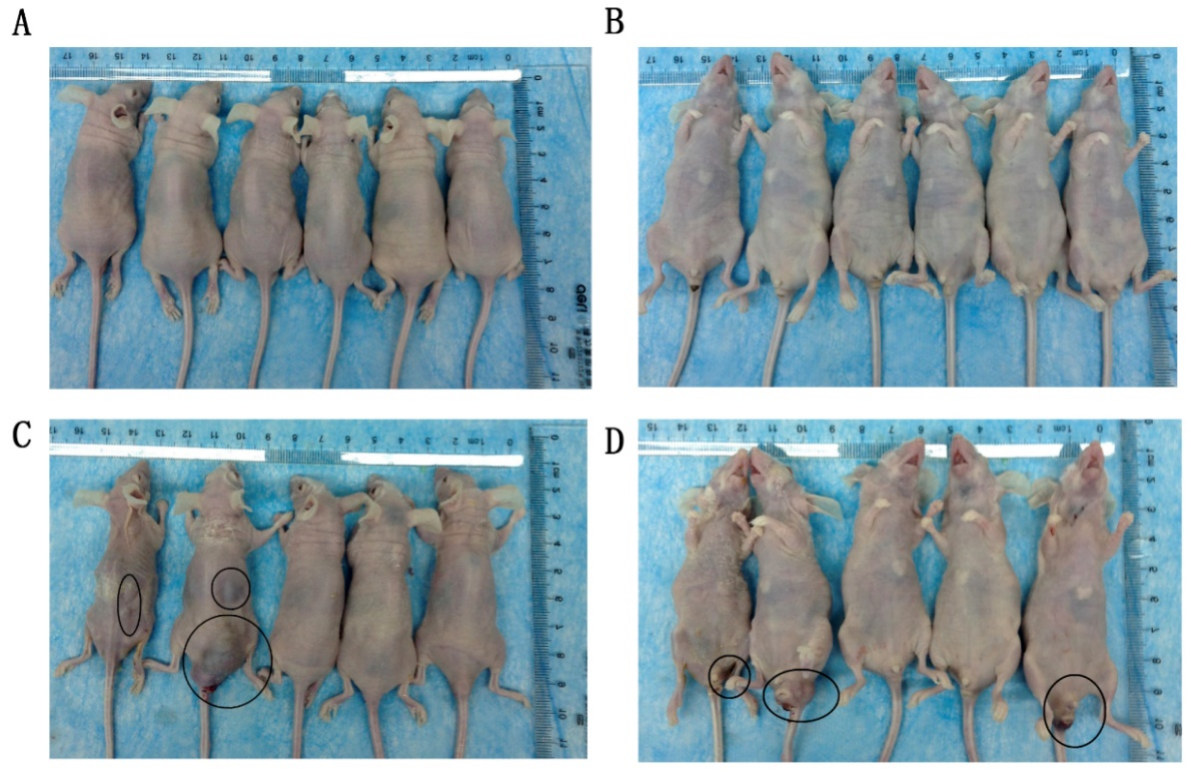
** Knockout of TGFBI suppresses tumor metastasis in vivo.** All mice that received HSC-3KO1 were normal and had no tumor-like protrusions on the body surface (A: dorsal surface, B: ventral surface). Mice injected with HSC-3 cells showed multiple tumor-like protrusions on the body surface, mainly located around the anus and the skin on the back of the neck (C: dorsal surface, D: ventral surface). Tumor-like nodules are indicated by black circles.

The effect of TGFBI on tumor metastasis in vivo was evaluated using a tail vein tumor metastasis model. At 60 days after cell injection, all mice that received HSC-3KO1 were normal, with no tumor-like protrusions found on their body surface (Fig. 5A, B). In contrast, mice injected with HSC-3 cells showed multiple tumor-like protrusions on their body surface, which were mainly located around the anus and the skin on the back of the neck (Fig. 5C, D). One of the mice died at a very early stage (on the 20^th^ day). The above results showed that TGFBI knockout could inhibit the metastatic ability of tumors in vivo.

### Identification of differentially expressed genes (DEGs) after TGFBI knockout in HSC-3 cell line

**Fig 6:**
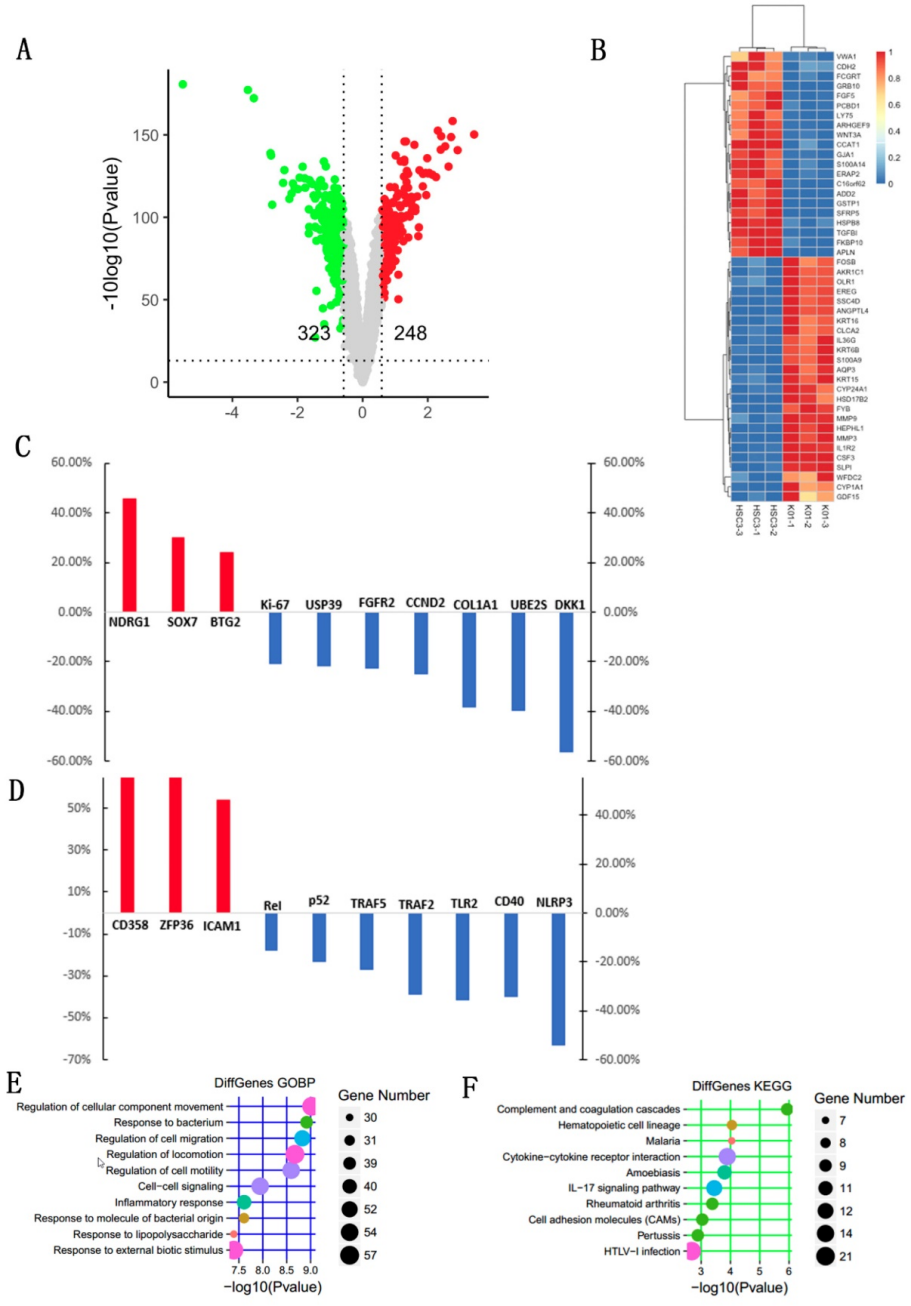
** Identification of DEGs and functional classification and annotation for the DEGs after TGFBI knockout in HSC-3.** A volcano plot for DEGs was created with cut-offs of p<0.05 and fold change >1.5 (A). A heat map for the DEGs was created with cut-offs of p<0.01 and fold change >3 (B). Some genes that had been reported to be related to OSCC but did not appear in the heat map also showed a change of expression after TGFBI knockout (C). Genes related to inflammation were differentially expressed after TGFBI knockout (D). The top 10 GO biological process terms of GO enrichment analysis (E). The top 10 pathways of KEGG pathway enrichment analysis (F).

We then compared the DEGs between the HSC-3KO1 and HSC-3 cell lines. The amount of sequencing data for each sample was approximately 6 Gb, which was sufficient for the whole-genome expression profile analysis. A total of 571 genes were differentially expressed after TGFBI knockout in HSC-3KO1, of which 248 genes were upregulated and 323 genes were downregulated. A volcano plot of the DEGs is shown in Fig. 6A. A heat map was also created for the genes that passed the cut-offs of p<0.01 and fold change >3.0 (Fig. 6B). Some genes that had been reported to be related to OSCC but did not appear in the heat map also showed a change of expression after TGFBI knockout (Fig. 6C). We also found that genes related to inflammation were differentially expressed after TGFBI knockout (Fig. 6D).

### Functional classification and annotation of DEGs identified by transcript sequencing

DEGs in HSC-3KO1 cells were subjected to KEGG and GO annotation in order to investigate their biological roles. The top 10 GO biological process terms are listed in Fig. 6E. The inclusion of the terms response to bacterium, response to lipopolysaccharide, and inflammatory response in this list suggests that TGFBI is related to the cellular responses to bacteria and their products and inflammation. The top 10 KEGG pathways are also listed in Fig. 6F. DEGs were significantly associated with malaria, amoebiasis, IL-17 signaling pathway, and rheumatoid arthritis, which are all pathogens or inflammation-related signaling pathways.

## Discussion

To the best of our knowledge, this is the first study focusing on the role of TGFBI in the pathogenesis of OSCC. In this study, we confirmed the expression of TGFBI in OSCC using both a bioinformatic approach and immunohistochemistry assays. We further explored the role of TGFBI in OSCC tumor growth and metastasis via TGFBI knockout in the HSC-3 cell line and a xenograft animal study. Our results revealed that TGFBI expression was higher in the OSCC tissue than in the normal tissue. Additionally, the survival rate of HNSCC patients with high TGFBI expression was lower than that of patients with low expression. Through analyzing our own clinical data, we also found that high TGFBI expression in OSCC was significantly associated with the T classification (*P*=0.037) and a higher clinical stage (*P*=0.007). These findings indicate that TGFBI may play an important role in the development of OSCC and could be regarded as a prognostic factor.

The effects of TGFBI on the biological behavior of OSCC were examined by knocking it out in HSC-3 cells using the CRISPR/Cas9 system. CRISPR/Cas9 is a newly developed gene editing tool that is highly specific and suitable for characterizing genetic elements and functions systematically 23, 24. In this study, we knocked out TGFBI in HSC-3 cells using two gRNAs against exon 3. RT-qPCR and western blotting confirmed that TGFBI had been knocked out successfully in both HSC-3KO1 and HSC-3KO3 cells, and thus that the cells were suitable for further study.

In the present study, knockout of TGFBI reduced cell proliferation and clone formation in vitro. Moreover, the HSC-3KO1 and HSC-KO3 cell-generated tumors grew slower than those generated by HSC-3 cells in vivo study. The decreased cell proliferation due to the inactivation of TGFBI was alleviated when TGFBI-knockout cells were cultured with the conditioned medium from the HSC-3 cells, suggesting that the extracellular TGFBI protein secreted by HSC-3 cells could regulate cell proliferation. The uncontrolled proliferation of cells is one of the main features of malignant tumors 25. The above results indicated that TGFBI exhibited oncogene-like characteristics and could promote the proliferation of cancer cells in OSCC.

As an extracellular matrix protein, TGFBI is closely related to tumor metastasis in different cancers 14, 26. Our results showed that the inactivation of TGFBI promoted cell migration and invasion in HSC-3 cells in vitro. However, a tail vein tumor metastasis model showed that TGFBI knockout could inhibit tumorigenicity and metastasis in vivo. We postulate that, although the inactivation of TGFBI would promote the migration and invasion of cells in vitro, the reduction of cell proliferation capacity would eventually bring the tumor into an inhibited state, and the cells transported a certain distance were unable to form tumors due to the weakening of their ability to proliferate in vivo. The findings also suggest that we need to consider all aspects of the target gene functions in the process of tumor gene targeted therapy.

For further exploration of the mechanism of action of TGFBI in the development of OSCC, we chose HSC-3 and HSC-3KO1 cells on which to perform transcriptional sequencing. Our results showed that TGFBI knockout significantly reduced the proliferation of HSC-3 cells. Transcriptional sequencing revealed a change of expression of many genes related to cancer cell proliferation. For example, COL1A1, FGFR2, UBE2S, CCND2, USP39, and APLN were downregulated after TGFBI knockout, which can inhibit the proliferation of OSCC cells 27-32. In addition, SOX7, BTG2, and NDRG1, which were regarded as tumor suppressor genes in OSCC 33-35, were upregulated after TGFBI knockout.

Many upregulated genes in the heat map have been reported to promote tumor metastasis, such as FOSB, OLR1, S100A9, MMP9, and ANGPTL4 36-40. This explains the changes in migration and invasion of TGFBI-knockout cells as observed in our study in vitro.

Through GO and KEGG enrichment analyses, we found that DEGs were closely related to the terms response to bacterium and inflammatory response. There were indeed many inflammation-related genes with a change of expression, such as p52, TLR2, TRAF5, and NLRP3. Long-term chronic inflammatory stimuli have been shown to have a close relationship with the development of cancer. Moreover, aberrant pathogenic or pathobiont microbial colonization is one of the important sources of chronic inflammation 5. The interaction between the pathogen and the host cell may cause an imbalance in the immune inflammatory response, thereby increasing the mutation rate of normal cells and triggering host cell proliferation and malignant transformation 41, 42. Studies found that *Porphyromonas gingivalis* and* Fusobacterium nucleatum* could promote the mutation of some tumor-related genes and induce OSCC, as revealed by cell experiments in vitro and animal model experiments in vivo 43, 44. Damage of the oral mucosal barrier or change of cell surface receptors may affect the cellular response to oral commensal bacteria, which can in turn trigger a corresponding inflammatory response. *Porphyromonas gingivalis* could upregulate TLR on the surface of oral epithelial cells and activate NF-κB and MAPK signaling pathways to promote inflammation 45. It has also been reported that TGFBI is a regulator of TLR-induced inflammation and participates in the process of endotoxin tolerance induced by low-dose LPS in peripheral blood mononuclear cells 46. Therefore, combining the results of GO and KEGG enrichment analyses, we speculated that TGFBI may be an important link between bacteria and tumors, which warrants further research.

## Conclusion

TGFBI overexpression promotes OSCC and is associated with poor prognosis in OSCC patients. TGFBI knockout can inhibit cell proliferation and metastasis in vivo. TGFBI may alter cell responses to bacteria, which causes an imbalance in the immune inflammatory response and promotes the development of OSCC. Exploring the role of TGFBI in OSCC may provide a new perspective on its clinical treatment and prognosis.

## Figures and Tables

**Table 1:** PCR primers used in this study

Gene	Forward primers (5´-3´)	Reverse primers (5´-3´)
TGFBI	TCCGCTAACCAGGATTTCATC	CCTCCGCTAACCAGGATTTCATC
S100A9	CACTCTGTGTGGCTCCTC	ATGGTCTCTATGTTGCGTTCC
ANGPTL4	AGACACAACTCAAGGCTCAG	CTCATGGTCTAGGTGCTTGTG
OLR1	GTTGAAGTTCGTGACTGCTTC	TGGAGAGTAAAGAAACTGAAGACC
CLCA2	AGTGGTCTGTTTAGTGCTGG	TCTCCTTTGCTGTCGAAACTG
KI-6	AAAAGAATTGAACCTGCGGAAG	AGTCTTATTTTGGCGTCTGGAG
DKK1	GTTACTGTGGAGAAGGTCTGTC	GTTCACTGCATTTGGATAGCTG
GRB10	CCATCCCCAATCCTTTTCCTG	CACTTTGCTTGTCCCATCTTC
APLN	TCTGGCTCTCCTTGACCG	CATTCCTTGACCCTCTG
β-actin	ACCTTCTACAATGAGCTGCG	CCTGGATAGCAACGTACATGG
sgRNA	GGAGGCAACTTAGTGGAGAGGGG	AGGCTCTATGCGAGGGGCTGAGG

**Table 2:** Correlation between TGFBI and clinicopathological parameters in OSCC

		TGFBI expression		
	N	Low expression (%)	High expression (%)	*P*-value
Total	52			
Gender				
Male	34	22(64.7)	12(35.3)	0.239
Female	18	8(44.4)	10(55.6)	
Age				
≥ 60	23	15(65.2)	8(34.8)	0.403
< 60	29	15(55.2)	14 (44.8)	
Histopathological grade				
Well	28	19(67.9)	9 (32.1)	0.534
Moderately and Poorly	24	11(45.8)	13(54.2)	
T classification				
T1+2	41	27(65.9)	14(34.1)	0.037
T3+4	11	3(27.3)	8(72.7)	
Lymph nodal status				
N0	41	26 (63.4)	15 (35.6)	0.169
N1/2/3	11	4 (36.4)	7(63.6)	
Stage				
I+II	35	25(71.4)	10(28.6)	0.007
III+IV	17	5(29.4)	12(70.6)	

## References

[1] Jemal A, Bray F, Center MM, Ferlay J, Ward E, Forman D (2011). Global cancer statistics. CA Cancer J Clin.

[2] Torre LA, Bray F, Siegel RL, Ferlay J, Lortet-Tieulent J, Jemal A (2015). Global cancer statistics, 2012. CA Cancer J Clin.

[3] Markopoulos AK (2012). Current aspects on oral squamous cell carcinoma. The open dentistry journal.

[4] Landskron G, De la Fuente M, Thuwajit P, Thuwajit C, Hermoso MA (2014). Chronic inflammation and cytokines in the tumor microenvironment. J Immunol Res.

[5] Elinav E, Nowarski R, Thaiss CA, Hu B, Jin C, Flavell RA (2013). Inflammation-induced cancer: crosstalk between tumours, immune cells and microorganisms. Nat Rev Cancer.

[6] Goertzen C, Mahdi H, Laliberte C, Meirson T, Eymael D, Gil-Henn H (2018). Oral inflammation promotes oral squamous cell carcinoma invasion. Oncotarget.

[7] He Y, Shao F, Pi W, Shi C, Chen Y, Gong D (2016). Largescale Transcriptomics Analysis Suggests Over-Expression of BGH3, MMP9 and PDIA3 in Oral Squamous Cell Carcinoma. PLoS One.

[8] SKONIER J, NEUBAUER M, Madisen L, BENNETT K, PLOWMAN GD, Purchio A (1992). cDNA cloning and sequence analysis of βig-h3, a novel gene induced in a human adenocarcinoma cell line after treatment with transforming growth factor-β. DNA and cell biology.

[9] Thapa N, Lee BH, Kim IS (2007). TGFBIp/betaig-h3 protein: a versatile matrix molecule induced by TGF-beta. Int J Biochem Cell Biol.

[10] Kim JE, Jeong HW, Nam JO, Lee BH, Choi JY, Park RW (2002). Identification of motifs in the fasciclin domains of the transforming growth factor-beta-induced matrix protein betaig-h3 that interact with the alphavbeta5 integrin. J Biol Chem.

[11] Bae JS, Lee SH, Kim JE, Choi JY, Park RW, Park JY (2002). beta ig-h3 supports keratinocyte adhesion, migration, and proliferation through alpha 3 beta 1 integrin. Biochemical and Biophysical Research Communications.

[12] Han B, Cai HL, Chen Y, Hu B, Luo HY, Wu YL (2015). The role of TGFBI (beta ig-H3) in gastrointestinal tract tumorigenesis. Molecular Cancer.

[13] Bhagirath D, Abrol N, Khan R, Sharma M, Seth A, Sharma A (2012). Expression of CD147, BIGH3 and Stathmin and their potential role as diagnostic marker in patients with urothelial carcinoma of the bladder. Clin Chim Acta.

[14] Ma C, Rong Y, Radiloff DR, Datto MB, Centeno B, Bao S (2008). Extracellular matrix protein betaig-h3/TGFBI promotes metastasis of colon cancer by enhancing cell extravasation. Genes Dev.

[15] Ozawa D, Yokobori T, Sohda M, Sakai M, Hara K, Honjo H (2016). TGFBI Expression in Cancer Stromal Cells is Associated with Poor Prognosis and Hematogenous Recurrence in Esophageal Squamous Cell Carcinoma. Ann Surg Oncol.

[16] Wen GY, Partridge MA, Li BY, Hong M, Liao WP, Cheng SK (2011). TGFBI expression reduces in vitro and in vivo metastatic potential of lung and breast tumor cells. Cancer Lett.

[17] Wen GY, Hong M, Li BY, Liao WP, Cheng SK, Hu BR (2011). Transforming growth factor-beta-induced protein (TGFBI) suppresses mesothelioma progression through the Akt/mTOR pathway. International Journal of Oncology.

[18] Li BY, Wen GY, Zhao YL, Tong J, Hei TK (2012). The role of TGFBI in mesothelioma and breast cancer: association with tumor suppression. Bmc Cancer.

[19] Ween MP, Oehler MK, Ricciardelli C (2012). Transforming Growth Factor-Beta-Induced Protein (TGFBI)/(beta ig-H3): A Matrix Protein with Dual Functions in Ovarian Cancer. International Journal of Molecular Sciences.

[20] Bertero L, Massa F, Metovic J, Zanetti R, Castellano I, Ricardi U (2018). Eighth Edition of the UICC Classification of Malignant Tumours: an overview of the changes in the pathological TNM classification criteria-What has changed and why?. Virchows Arch.

[21] JJ P, Reichart PA, CJ S, I vdW (1997). Histological typing of cancer and precancer of the oral mucosa,2nd edition. World health organization (WHO). Springer-Verlag.

[22] Fengkai X, Jie G, Lin W (2018). Up-regulation Of EIF3e Is Associated With The Progression Of Esophageal Squamous Cell Carcinoma And Poor Prognosis In Patients. Journal of Cancer.

[23] Zhang F, Wen Y, Guo X (2014). CRISPR/Cas9 for genome editing: progress, implications and challenges. Hum Mol Genet.

[24] Jubair L, McMillan NAJ (2017). The Therapeutic Potential of CRISPR/Cas9 Systems in Oncogene-Addicted Cancer Types: Virally Driven Cancers as a Model System. Mol Ther Nucleic Acids.

[25] Sebolt-Leopold JS, Herrera R (2004). Targeting the mitogen-activated protein kinase cascade to treat cancer. Nat Rev Cancer.

[26] Lauden L, Siewiera J, Boukouaci W, Ramgolam K, Mourah S, Lebbe C (2014). TGF-beta-Induced (TGFBI) Protein in Melanoma: A Signature of High Metastatic Potential. Journal of Investigative Dermatology.

[27] He B, Lin X, Tian F, Yu W, Qiao B (2018). MiR-133a-3p Inhibits Oral Squamous Cell Carcinoma (OSCC) Proliferation and Invasion by Suppressing COL1A1. J Cell Biochem.

[28] Yang X, Ruan H, Hu X, Cao A, Song L (2017). miR-381-3p suppresses the proliferation of oral squamous cell carcinoma cells by directly targeting FGFR2. Am J Cancer Res.

[29] Yoshimura S, Kasamatsu A, Nakashima D, Iyoda M, Kasama H, Saito T (2017). UBE2S associated with OSCC proliferation by promotion of P21 degradation via the ubiquitin-proteasome system. Biochem Biophys Res Commun.

[30] Zeng Q, Tao X, Huang F, Wu T, Wang J, Jiang X (2016). Overexpression of miR-155 promotes the proliferation and invasion of oral squamous carcinoma cells by regulating BCL6/cyclin D2. Int J Mol Med.

[31] Li KY, Zhang J, Jiang LC, Zhang B, Xia CP, Xu K (2016). Knockdown of USP39 by lentivirus-mediated RNA interference suppresses the growth of oral squamous cell carcinoma. Cancer Biomark.

[32] Heo K, Kim YH, Sung HJ, Li HY, Yoo CW, Kim JY (2012). Hypoxia-induced up-regulation of apelin is associated with a poor prognosis in oral squamous cell carcinoma patients. Oral Oncology.

[33] Oh KY, Hong KO, Huh YS, Lee JI, Hong SD (2017). Decreased expression of SOX7 induces cell proliferation and invasion and correlates with poor prognosis in oral squamous cell carcinoma. J Oral Pathol Med.

[34] Lee JC, Chung LC, Chen YJ, Feng TH, Chen WT, Juang HH (2015). Upregulation of B-cell translocation gene 2 by epigallocatechin-3-gallate via p38 and ERK signaling blocks cell proliferation in human oral squamous cell carcinoma cells. Cancer Lett.

[35] Lee JC, Chung LC, Chen YJ, Feng TH, Juang HH (2014). N-myc downstream-regulated gene 1 downregulates cell proliferation, invasiveness, and tumorigenesis in human oral squamous cell carcinoma. Cancer Lett.

[36] Barrett CSX, Millena AC, Khan SA (2017). TGF- Effects on Prostate Cancer Cell Migration and Invasion Require FosB. Prostate.

[37] Wang B, Zhao H, Zhao L, Zhang Y, Wan Q, Shen Y (2017). Up-regulation of OLR1 expression by TBC1D3 through activation of TNFalpha/NF-kappaB pathway promotes the migration of human breast cancer cells. Cancer Lett.

[38] Lim SY, Yuzhalin AE, Gordon-Weeks AN, Muschel RJ (2016). Tumor-infiltrating monocytes/macrophages promote tumor invasion and migration by upregulating S100A8 and S100A9 expression in cancer cells. Oncogene.

[39] Bedal KB, Grassel S, Oefner PJ, Reinders J, Reichert TE, Bauer R (2014). Collagen XVI induces expression of MMP9 via modulation of AP-1 transcription factors and facilitates invasion of oral squamous cell carcinoma. PLoS One.

[40] Huang Z, Xie J, Lin S, Li S, Huang Z, Wang Y (2016). The downregulation of ANGPTL4 inhibits the migration and proliferation of tongue squamous cell carcinoma. Arch Oral Biol.

[41] Guven Maiorov E, Keskin O, Gursoy A, Nussinov R (2013). The structural network of inflammation and cancer: merits and challenges. Semin Cancer Biol.

[42] Tribble GD, Kerr JE, Wang BY (2013). Genetic diversity in the oral pathogen Porphyromonas gingivalis: molecular mechanisms and biological consequences. Future Microbiol.

[43] Binder Gallimidi A, Fischman S, Revach B, Bulvik R, Maliutina A, Rubinstein AM (2015). Periodontal pathogens Porphyromonas gingivalis and Fusobacterium nucleatum promote tumor progression in an oral-specific chemical carcinogenesis model. Oncotarget.

[44] Geng F, Liu J, Guo Y, Li C, Wang H, Wang H (2017). Persistent Exposure to Porphyromonas gingivalis Promotes Proliferative and Invasion Capabilities, and Tumorigenic Properties of Human Immortalized Oral Epithelial Cells. Front Cell Infect Microbiol.

[45] Groeger S, Jarzina F, Domann E, Meyle J (2017). Porphyromonas gingivalis activates NFkappaB and MAPK pathways in human oral epithelial cells. BMC immunology.

[46] Yang Y, Sun HX, Li XY, Ding Q, Wei PJ, Zhou JG (2015). Transforming Growth Factor Beta-Induced Is Essential for Endotoxin Tolerance Induced by a Low Dose of Lipopolysaccharide in Human Peripheral Blood Mononuclear Cells. Iran J Allergy Asthm.

